# Involvement of normalized NMDA receptor and mTOR-related signaling in rapid antidepressant effects of Yueju and ketamine on chronically stressed mice

**DOI:** 10.1038/srep13573

**Published:** 2015-08-28

**Authors:** Juanjuan Tang, Wenda Xue, Baomei Xia, Li Ren, Weiwei Tao, Chang Chen, Hailou Zhang, Ruyan Wu, Qisheng Wang, Haoxin Wu, Jinao Duan, Gang Chen

**Affiliations:** 1 Center for Translational Systems Biology and Neuroscience, School of Basic Biomedical Science, Nanjing University of Chinese Medicine, Nanjing 210023, China; 2 Key Laboratory of Integrative Biomedicine of Brain Diseases, School of Basic Biomedical Science, Nanjing University of Chinese Medicine, Nanjing 210023, China; 3 Physiology Research Section, School of Basic Biomedical Science, Nanjing University of Chinese Medicine, Nanjing 210023, China; 4 First Clinical Medical College, Nanjing University of Chinese Medicine, Nanjing 210023, China

## Abstract

Yueju, a Traditional Chinese Medicine formula, exhibited fast-onset antidepressant responses similar to ketamine. This study focused on assessing the rapid and persistent antidepressant efficacy of Yueju and ketamine in chronically stressed mice and its association with alternations in prefrontal N-methyl-D-aspartate (NMDA) receptor and mammalian target of rapamycin (mTOR)-related activity. Chronic mild stress (CMS) led to deficits in sucrose preference test (SPT), forced swim test, tail suspension test, and novelty-suppressed feeding test, which were improved differently by acute Yueju or ketamine administration. The improvement in SPT started as soon as 2 hours post Yueju and ketamine but lasted for 6 days only by Yueju. Body weight was regained by Yueju more than ketamine at post-drug administration day (PAD) 6. CMS decreased phosphorylation of the mTOR effectors 4E-BP1 and p70S6K, their upstream regulators ERK and Akt, and downstream targets including synaptic protein GluR1. Yueju or ketamine reversed these changes at PAD 2, but only Yueju reversed phosphor-Akt at PAD 6. CMS selectively and lastingly increased NMDA receptor subunit NR1 expression, which was reversed by ketamine or Yueju at PAD 2 but only by Yueju at PAD 6. These findings suggest that NR1 and Akt/mTOR signaling are important therapeutic targets for depression.

Major depressive disorder (MDD), a serious mental disorder, is the leading cause of disability and a major contributor to disease burden in the world’s population^1^. Sufficient evidence indicates progressive structural changes in the central nervous system are associated with the persistent clinical signs and symptoms of MDD^2^. Therefore, it is very important to treat MDD as quickly and effectively as possible. Monoamine-based serotonin selective reuptake inhibitors (SSRIs) represent the first-line antidepressants, however, only two thirds of MDD patients respond to them. Furthermore, the therapeutic effect of an SSRI is achieved over several weeks^3^. Therefore, development of fast-onset and effective antidepressants is urgently needed^4^.

Recent studies demonstrate that ketamine, a glutamatergic N-methyl-D-aspartate receptor (NMDA-R) antagonist, exhibits fast-onset and long-lasting antidepressant effects^5^,^6^,^7^. Symptoms of depression were attenuated from 2 hours to several days after a single low dose of ketamine to MDD patients^8^. This is similar to the actions of ketamine in rodent models of depression^9^,^10^. The clinical wide use of ketamine is challenged by the potential toxic and addictive effects of ketamine^11^. Subsequently, a number of fast-acting antidepressants have been identified, including NMDA 2B subunit antagonists^10^, NMDAR glycine-site functional partial agonists^12^, mGluR_2/3_ antagonists^13^ and Yueju^14^.

Several studies suggest that ketamine and other fast-acting antidepressants, mediated by glutamate and/or neurotrophic receptors, stimulate the mammalian target of rapamycin (mTOR) pathway in the prefrontal cortex (PFC)^6^,^15^, leading to transient activation of the downstream effectors, 4E-BP1 and p70S6K, which regulate gene expression and protein synthesis. Stimulation of mTOR signaling is quickly followed by increased expression of synaptic proteins such as PSD-95 and synapsin-1 and increased spine synapses^6^. Inhibition of mTOR, or ERK and Akt activation, upstream of 4E-BP1 and p70S6K, blocks the synaptic protein synthesis and antidepressant effects of ketamine^6^. These observations suggest that rapid changes in synaptic protein contents induced by mTOR activation may contribute to the fast-acting antidepressant effects of ketamine and similar drugs^5^. However, the findings were mostly based on changes in the PFC of non-stressed animals, in which mTOR and its downstream effectors were activated but then shortly returned to baseline. Recent studies have suggested that mTOR signaling is compromised in depressed patients and animal models of depression^16^,^17^. These findings raise the possibility that mTOR signal pathways are potential therapeutic targets for antidepressant actions in depressed subjects.

Many of the current fast-acting antidepressants down-regulate glutamate neurotransmission. Glutamate released from presynaptic neurons interacts with postsynaptic glutamate receptors, including NMDA, kainate, and AMPA. Blockade of AMPA receptors (AMPAR) blunts ketamine’s antidepressant effects in mice and rats^6^,^9^,^18^, whereas enhanced AMPAR signaling facilitates the effects^19^. An increasing number of studies indicate that dysregulation of NMDA and AMPA receptor expression and activity by stress may contribute to mental disorders including depression^20^,^21^,^22^. Based on pharmacological studies, an increase in the AMPA/NMDA receptor ratio is at least partially responsible for antidepressant responses^23^,^24^. An increase in AMPA to NMDA receptor density (increased AMPAR/NMDAR ratio) has been observed after chronic ketamine treatment^25^. To date, evidence supports that acute ketamine leads to lasting up-regulated expression of AMPA receptor subunit GluR1^6^. An evaluation of NMDA receptor subunits would provide a better understanding of the glutamate neurotransmission mechanism of rapid antidepressant responses, particularly in depressed subjects who may have abnormal NMDA and/or AMPA functions.

Chronic mild stress (CMS) is a well-validated and commonly used model to mimic clinic depression^26^. In a CMS model, a relatively extended SSRI treatment is needed before an antidepressant effect is observed. In contrast, CMS has been used to demonstrate a rapid-onset antidepressant effect after a single dose of ketamine^10^. Here, we tested whether alterations in NMDA receptors and associated mTOR signaling in the PFC are part of the pathology of depression and are part of the therapeutic responses to antidepressant actions of ketamine and Yueju using a CMS mouse model. Yueju, a herb medicine formulated 800 years ago to treat the disorders derived from stress, or Qi stagnation symptoms, is an effective natural agent for depression treatment and contains multiple antidepressant components^27^,^28^. A recent study showed the rapid antidepressant potential of ethanol extract of Yueju using acute or subacute behavioral paradigms^14^. We employed a CMS model in mice, and performed a comprehensive characterization of depression-like or anxiety-like behavioral changes after a single administration of ketamine or Yueju. The time course changes in antidepressant efficacy of ketamine and Yueju were also monitored, and fluoxetine, an SSRI, was also used to compare for rapid antidepressant effects in the CMS mouse model. Furthermore, expression of upstream and downstream targets of mTOR and NMDA receptor subunits in the PFC were examined at different time points when ketamine and Yueju showed similar or differential antidepressant responses.

## Results

### Rapid and differential long-lasting Antidepressant effects of ketamine and Yueju on CMS mice

**Figure 1:**
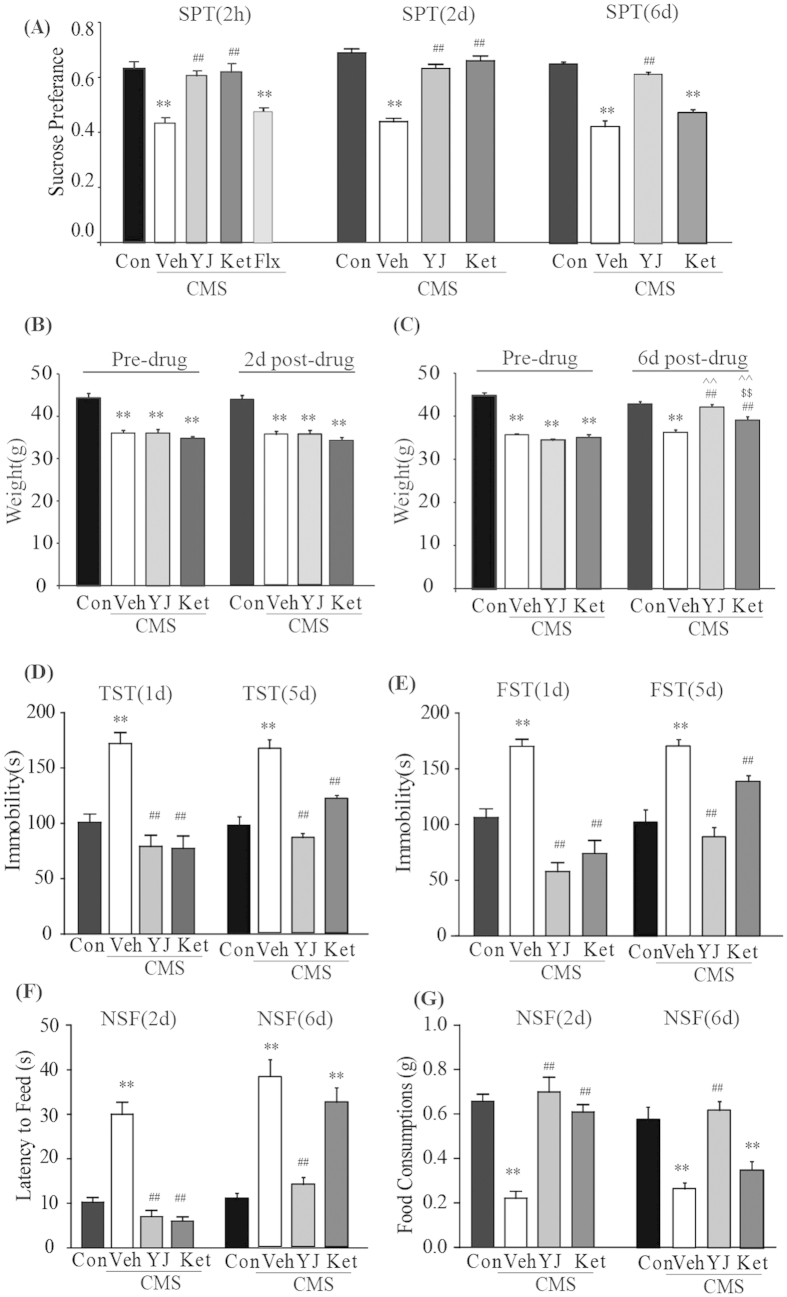
Behavioral effects at different time points after a single ketamine and Yueju treatment in CMS mice. Control animals (Con) received vehicle treatment, and animals exposed to CMS received a single administration of vehicle (Veh), Yueju (YJ) or ketamine (Ket). (**A**) Sucrose preference test at 2 hours, 2 days and 6 days after drug administration, respectively. ANOVA, 2 hours, F(4,39) = 16.387, p < 0.01; 2 days, F(3,31) = 55.55, p < 0.01; 6 days, F(3,37) = 50.78, p < 0.01. (**B**,**C**) Body weight changes at 2 days and 6 days post drug administration. ANOVA with repeated measurement, 2 days, Time: F(1,31) = 33.890, p = 0.000, Group: F(3,31) = 29.337, p = 0.000, Time*Group: F(3,31) = 0.422, p = 0.739; 6 days, Time: F(1,34) = 72.432, p = 0.000, Group: F(3,34) = 45.658. p = 0.000, Time*Group: F(3,34) = 29.912, p = 0.000. (**D**) The immobility time in the TST at 1 day and 5 days post drug administration. ANOVA, 1 day, F(3,31) = 18.227, p < 0.01; 5 days, F(3,37) = 35.09, p < 0.01. (**E**) The immobility time in the FST 1 day and 5 days after drug administration. ANOVA, 1 day, F(3,31) = 24.995, p < 0.01; 5 days, F(3,37) = 21.49, p < 0.01. (**F**) Latency to feed during the NSF test at 2 days and 6 days post drug administration. ANOVA, 2 days, F(3,31) = 41.395, p < 0.01; 6 days, F(3,37) = 25.13, p < 0.01, and (**G**) Total amount of food consumed during the NSF test at 2 days and 6 days post drug administration. ANOVA, 2 days, F(3,31) = 31.341, p < 0.01; 6 days, F(3,37) = 16.476, p < 0.01. **p < 0.01, compared with control group; ##p < 0.01, compared with vehicle group; $$p < 0.01, compared with ketamine group; ^^p < 0.01, compared with pre-drug treatment level. n = 7−10.

Three independent cohorts of animals were used to test the rapid and long-lasting antidepressant effects of ketamine and Yueju on CMS mice, measured with sucrose preference test, tail suspension test, forced swim test, novelty-suppressed feeding test, open field test, as well as body weight (Table 1, Fig. 1). CMS exposure resulted in a significant decrease in sucrose preference in all three cohorts (p < 0.01 vs naïve). As shown in Fig. 1A, the reduction in sucrose preference was ameliorated as soon as 2 hours following a single administration of ketamine or Yueju (both p < 0.01, vs vehicle), but not a single dose of fluoxetine (p = 0.478) (ANOVA, F(4,39) = 16.387, p < 0.01). Reversal of deficits in sucrose preference was evident for both ketamine and Yueju at post-drug administration (PAD) 2 (both p < 0.01 vs vehicle) (ANOVA, F(3,31) = 55.55, p < 0.01) but only for Yueju at PAD 6 (p < 0.01 vs vehicle) (ANOVA, F(3,37) = 50.78, p < 0.01) (Fig. 1A). CMS significantly reduced the body weight gain at PAD 2 (repeated ANOVA, Time, F(1, 31) = 33.890, p = 0.000, Group: F(3,31) = 29.337, p = 0.000, Time*Group: F(3,31) = 0.422, p = 0.739) and PAD 6 (repeated ANOVA, Time, F(1,34) = 72.432, p = 0.000, Group: F(3,34) = 45.658, p = 0.000, Time*Group: F(3,34) = 29.912, p = 0.000) in the CMS mice (both p < 0.01, vs naive). Yueju or ketamine treatment did not change the weight at PAD 2. However, both Yueju and ketamine significantly increased body weight at PAD 6 days compared with CMS group (both p < 0.01), and Yueju-treated mice gained more weight than ketamine-treated mice (p < 0.01) (Fig. 1B,C).

Both ketamine and Yueju showed lasting antidepressant effects in the behavioral despair paradigm in chronically stressed mice. At PAD 1, CMS mice displayed a significant increase in immobility time during the TST (Fig. 1D, p < 0.01 vs naïve) and FST (Fig. 1E, p < 0.01 vs naïve). Both Yueju and ketamine reversed the CMS-induced increase of immobility time in the TST (both p < 0.01, vs vehicle) (ANOVA, F(3,31) = 18.227) and FST (both p < 0.01, vs vehicle group) (ANOVA, F(3,31) = 24.995, p < 0.01). The effects on TST (ANOVA, F(3,37) = 35.09, p < 0.01) or FST (ANOVA, F(3,37) = 21.49, p < 0.01) were still significant for both ketamine (TST, p < 0.01; FST, p < 0.01, vs vehicle) and Yueju (TST, p < 0.01; FST, p < 0.01, vs vehicle) at PAD 5.

In the NSF test, at PAD 2, CMS increased the latency to feed (p < 0.01 vs naïve group) (ANOVA, F(3,31) = 41.395, p < 0.01) (Fig. 1F,G). The CMS exposed mice also consumed significantly less food (p < 0.01 vs naïve) (ANOVA, F(3,31) = 31.341, p < 0.01). These changes were reversed by ketamine or Yueju (both p < 0.01, vs vehicle group) at PAD 2. At PAD 6, the effects remained significant only for Yueju (latency to feed, p < 0.01, vs vehicle; food consumed, p < 0.01, vs vehicle), but not ketamine (latency to feed, ANOVA, F(3,37) = 25.13, p < 0.01; food consumed, ANOVA, F(3,37) = 16.476, p < 0.01). In the open field test, there was no effect of CMS on either total distance traveled, a measurement of locomotor (PAD 1, ANOVA, F(3,31) = 1.472, p = 0.244; PAD 5, ANOVA, F(3,37) = 1.607, p = 0.206), or the time spent in the central zone (PAD 1, ANOVA, F(3,31) = 1.194, p = 0.33; PAD 5, ANOVA, F(3,37) = 1.032, p = 0.391), a measurement of anxiety, indicating that this CMS procedure did not alter open field behavior (Supplement 2). These results identified 2 hours as the fast-onset time as well as 5 or 6 days as the long-lasting time periods of antidepressant effects of ketamine or Yueju in the CMS mice, respectively.

### Ketamine and Yueju treatment improved expressions of mTOR effectors in the PFC of CMS mice

**Figure 2:**
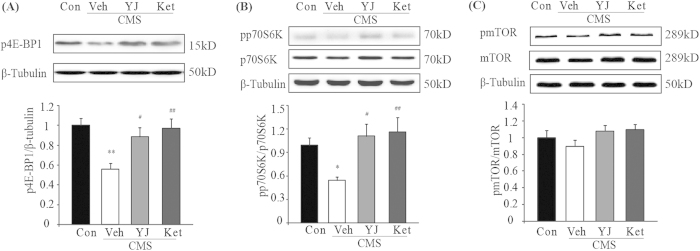
Alternations of activation of downstream effectors of mTOR signaling in PFC of CMS mice at 2 days post ketamine and Yueju administration. (**A**) Phosphorylation of 4E-BP1 level. ANOVA, F(3,20) = 6.138, p < 0.01. (**B**) Phosphorylated p70S6K (pp70S6K) normalized to total p70S6K, ANOVA, F(3,20) = 4.042, p < 0.05. (**C**) phosphorylated mTOR (p-mTOR) normalized to total mTOR, ANOVA, F(3,20) = 1.443, p = 0.265. *p < 0.05, **p < 0.01, compared with control group. #p < 0.05, ##p < 0.01, compared with vehicle group, and n = 5−7.

Activation of mTOR in the PFC is required for a fast-onset and long-lasting antidepressant effect^6^,^29^. Phosphorylated forms of eukaryotic initiation factor 4E binding protein 1 (4E-BP1), and p70S6 kinase (p70S6K) are the two mTOR effectors. As shown in Fig. 2, at PAD 2, the CMS procedure significantly decreased phosphorylation of 4E-BP1 (ANOVA, F(3,20) = 6.138, p < 0.01) and p70S6K (ANOVA, F(3,20) = 4.042, p < 0.05) in the PFC of mice (p < 0.01, p < 0.05 respectively, vs naïve), which were reversed by Yueju and ketamine treatment (both p < 0.05, vs vehicle group) (Fig. 2A,B). However, the CMS procedure did not have a significant effect on mTOR protein expression or phosphorylation, nor did Yueju or ketamine (ANOVA, F(3,20) = 1.443, p = 0.265) at 2 days post-drug administration (Fig. 2C).

### Ketamine and Yueju improved ERK and Akt signaling in the PFC of CMS mice

**Figure 3:**
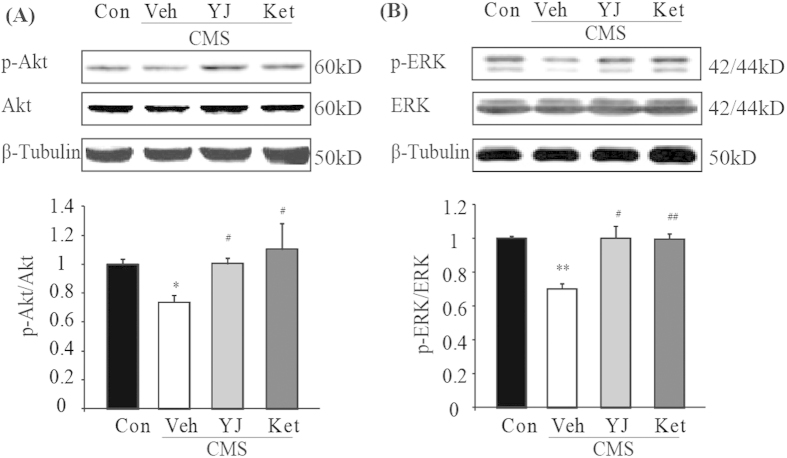
Alternations of activation of ERK1/2 and Akt in the PFC of CMS mice at 2 days post ketamine and Yueju administration. (**A**) Densitometric analysis of phosphorylated Akt normalized to total Akt, ANOVA, F(3,20) = 4.042, p < 0.05. and (**B**) phosphorylated ERK1/2 normalized to total ERK1/2, ANOVA, F(3,20) = 1.443, p < 0.05. *p < 0.05, **p < 0.01, compared with control group. #p < 0.05, ##p < 0.01, compared with vehicle group, and n = 5−7.

Activation of Akt and ERK1/2, which are required for lasting and fast antidepressant effects^6^, stimulate 4E-BP1 or p70S6K activation in an mTOR-dependent or mTOR-independent manner. As the activation of mTOR was unaltered by CMS or rapid antidepressant treatment, we tested whether CMS led to deficits in Akt or ERK1/2 activation and whether ketamine or Yueju reversed the deficits, in a pattern consistent with 4E-BP1 or p70S6K at PAD 2. As shown in Fig. 3, the CMS exposure resulted in a significant decrease in the phosphorylation of Akt (ANOVA, F(3,20) = 4.042, p < 0.05) and ERK1/2 (ANOVA, F(3,20) = 1.443, p < 0.05) in the PFC of mice (p < 0.05, p < 0.01 respectively, vs naïve), both of which were reversed by Yueju or ketamine treatment (both p < 0.05, vs vehicle) (Fig. 3A,B).

### Ketamine and Yueju treatment normalized the AMPA and NMDA subunit expressions in the PFC of CMS mice

**Figure 4:**
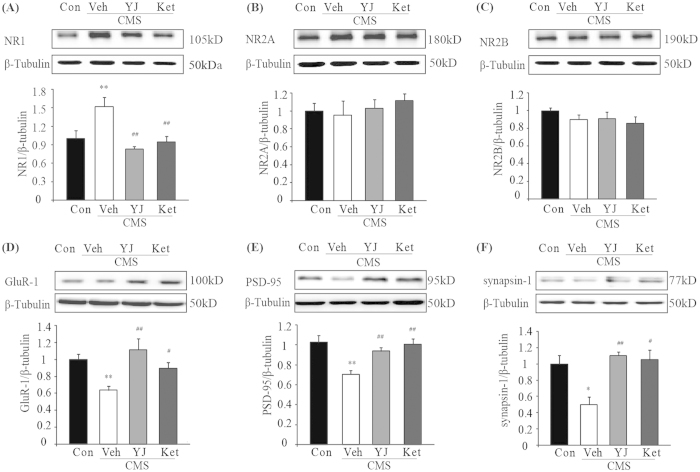
Alternations of synaptic proteins in the PFC of mice by CMS as well as ketamine and Yueju treatment. The treatment effects on protein expression levels in the PFC were determined with western blotting at 2 days post a single administration of Yueju or ketamine. (**A**) NR1, ANOVA, F(3,18) = 7.243, p < 0.01. (**B**) NR2A, ANOVA, F(3,18) = 0.391, p = 0.761. (**C**) NR2B, ANOVA, F(3,18) = 0.919, p = 0.454. (**D**) GluR-1 , ANOVA, F(3,18) = 5.896, p < 0.01. (**E**) PSD-95, ANOVA, F(3,18) = 7.719, p < 0.01. and (**F**) synapsin-1, ANOVA, F(3,18) = 9.36, p < 0.01. **p < 0.01, compared with control group. #p < 0.05, ##p < 0.01, compared with vehicle group, and n = 4−5.

NMDA receptors are implicated in the pathophysiology and treatment of depression^30^. Therefore, we tested whether ketamine or Yueju altered NMDA receptors in the PFC of chronically stressed mice. Functional NMDA receptors are tetramers, comprised of two obligatory NR1 subunits, and one NR2A and NR2B subunit in the cortex. As shown in Fig. 4, at PAD 2, CMS increased significantly expression of NR1 (p < 0.01, vs naïve). This increase was significantly attenuated by ketamine (Fig. 4A, p < 0.01, vs vehicle) and Yueju (p < 0.01, vs vehicle) (ANOVA, F(3,18) = 7.243, p < 0.01). The CMS procedure did not alter the expression of the other two subunits of NMDA receptors, NR2A (ANOVA, F(3,18) = 0.391, p = 0.761) and NR2B (ANOVA, F(3,18) = 0.919, p = 0.454), neither did Yueju or ketamine treatment (Fig. 4B,C). In contrast, chronic mild stress significantly reduced the expression of the AMPA receptor subunit GluR1 (Fig. 4D, p < 0.01, vs naïve) as well as other synaptic proteins PSD-95 (Fig. 4E, p < 0.01, vs naïve) and synapsin-1 (Fig. 4F, p < 0.01, vs naïve). At PAD 2, Yueju reversed the decreased expression of GluR1 (ANOVA, F(3,18) = 5.896, p < 0.01) (Fig. 4D, p < 0.01, vs vehicle), PSD-95 (ANOVA, F(3,18) = 7.719, p < 0.01) (Fig. 4E, p < 0.05, vs vehicle) and synapsin-1 (ANOVA, F(3,18) = 9.36, p < 0.01) (Fig. 4F, p < 0.05, vs vehicle). Ketamine showed similar effects (p < 0.05, p < 0.01, p < 0.05, respectively, vs vehicle).

### Signaling associated with antidepressant effects at 6 days post administration

**Figure 5:**
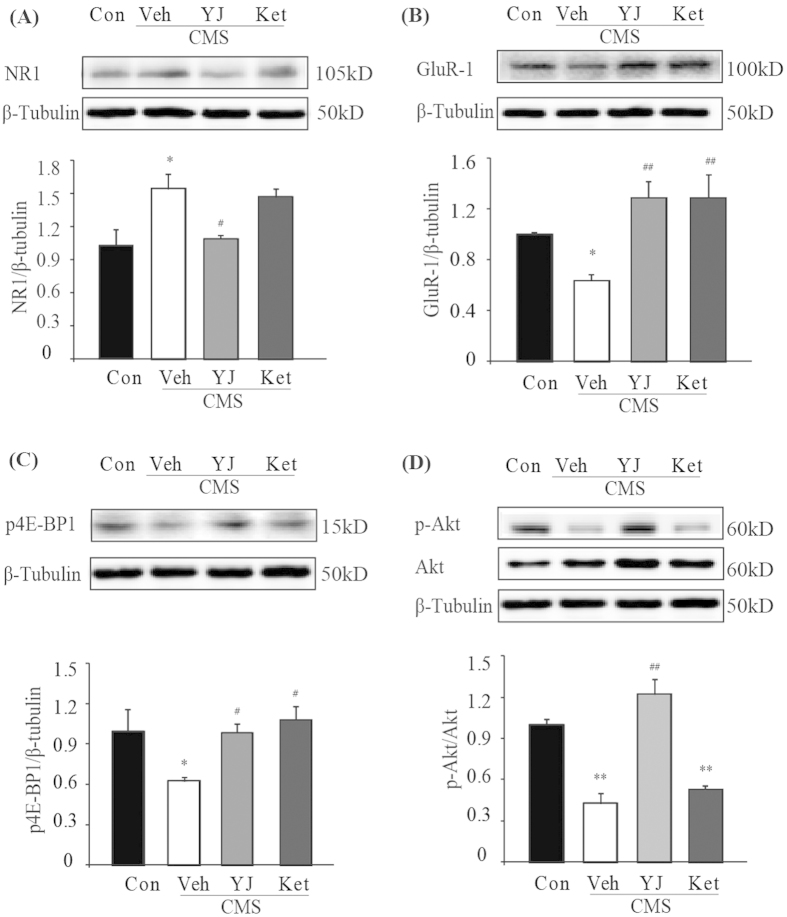
Alternations of synaptic proteins and mTOR signaling in the PFC of CMS mice at 6 days post ketamine and Yueju administration. The treatment effects on protein expression levels in the PFC were determined with western blotting. (**A**) NR1 ANOVA, F(3,15) = 6.1, p < 0.01. (**B**) GluR-1, ANOVA, F(3,15) = 7.2, p < 0.01. (**C**) Phosphorylation of 4E-BP1 level. ANOVA, F(3,15) = 3.937, p < 0.05. (**D**) phosphorylated Akt normalized to total Akt, ANOVA, F(3,15) = 28.557, p < 0.01. *p < 0.05, **p < 0.01, compared with control group. #p < 0.05, ##p < 0.01, compared with vehicle group, and n = 4.

As Yueju, but not ketamine, remained capable of exhibiting antidepressant effects at PAD 6, we thus assessed the signaling that may underlie the behavioral differences at this time point. Only NR1 and phosphorylation of Akt were reversed by Yueju but not ketamine in the CMS mice (Fig. 5). Expression of NR1 (ANOVA, F(3,15) = 6.1, p < 0.01) and GluR1 (ANOVA, F(3,15) = 7.2, p < 0.01) both continued to be significantly increased and decreased, respectively, by CMS (both p < 0.05, vs naïve). The increased expression of NR1 was significantly attenuated by Yueju (Fig. 5A, p < 0.05, vs vehicle), but not ketamine (p > 0.05, vs vehicle). In contrast, both ketamine and Yueju reversed the decreased expression of GluR1 (Fig. 5B, both p < 0.01, vs vehicle). At this time, CMS or drug treatments no longer showed effects on expressions of PSD-95 (ANOVA(3,15) = 0.718, p = 0.56) or synapsin-1 (ANOVA(3,15) = 0.323, p = 0.809).

For activities related to mTOR signaling, phosphorylation of 4E-BP1 (ANOVA, F(3,15) = 3.937, p < 0.05) continued to be reduced at 6 days post-drug administration (p < 0.05, vs naïve), which was reversed by both Yueju and ketamine treatment (both p < 0.05, vs vehicle) (Fig. 5C). In contrast, phosphorylation of p70S6K recovered and drug treatments no longer showed a significant effect (ANOVA, F(3,15) = 1.063, p = 0.401). Interestingly, phosphorylation of Akt (ANOVA, F(3,15) = 28.557, p < 0.01) remained deficient in CMS mice (p < 0.01, vs naïve), which was reversed by Yueju (p < 0.01, vs vehicle), but not ketamine treatment (p > 0.05, vs vehicle) (Fig. 5D). There was, however, no main effect on phosphorylation of ERK1/2 (ANOVA, F(3,15) = 0.039, p = 0.989).

## Discussion

Fast acting antidepressants that target glutamatergic receptors represent an important breakthrough in the therapy for depression^31^. Previous studies have shown the important role of mTOR and related signaling in rapid antidepressant effects in non-stressed animals^6^. The present study focused on examining changes in glutamatergic receptors and mTOR signaling affected by chronic stress and therapeutic mechanisms for two distinct rapid-acting antidepressants, ketamine and Yueju. Both ketamine and Yueju rapidly reversed the depression-like responses in the CMS exposed mice, and remained effective at 5 and 6 days post-drug administration, respectively. CMS compromised ERK/Akt signaling and downstream mTOR effectors in the PFC of CMS mice, which were resumed by Yueju or ketamine treatment. The normalization of Akt signaling was particularly associated with the Yueju’s lasting antidepressant effect. Both ketamine and Yueju quickly reversed the down-regulated expressions of the GluR1 subunit and selectively up-regulated expression of NR1 subunit in the CMS mice, whereas Yueju showed a longer duration in reversing NR1 level. These effects may increase the AMPAR/NMDAR ratio for antidepressant responses of Yueju and ketamine. Taken together, a rapid and lasting remedy of mTOR-related signaling and glutamatergic function in the PFC may, at least in part, be mechanistically linked with rapid and lasting antidepressant actions in depression-like subjects.

CMS is a well-developed and widely-used rodent model of depression^32^. A variety of procedures have been employed for CMS in rats and mice. Here, we used a CMS procedure leading to a full spectrum of depression-like and/or anxiety-like symptoms in mice, including decreased sucrose preference, increased immobility time in both TST and FST, and increased latency for eating and decreased food consumption in the NSF test, consistent with findings from some previous reports^33^,^34^. Furthermore, we for the first time demonstrated that a single administration of ketamine or Yueju improved the body weight gain at 6 days post-treatment, with a more pronounced restoration by Yueju. Body weight change is an important clinic symptom of depression and the normalization of body weight may represent part of functional recovery from depression. The present study, using a mouse chronic model of depression validated with multiple measurements of depression-like responses, suggests rapid antidepressant effects after a single dose of ketamine or Yueju. A pilot clinical study supported rapid antidepressant effects of Yueju on MDD patients (Wu and Chen, unpublished observations).

Additionally, the present study characterized the onset and duration of rapid antidepressant effects in chronically stressed mice. This is the first demonstration in CMS mice of a rapid antidepressant response (within 2 hours) for acute ketamine or Yueju, but not fluoxetine, a typical SSRI. Two hours post-ketamine is also a time point when MDD patients started to experience an improvement in mood induced by ketamine treatment^35^. Garcia *et al.* assessed the early time points for antidepressant responses in chronically stressed rats, but did not detect a significant effect of ketamine on sucrose intake 1 hour after administration^33^. It takes about 2 hours for ketamine to promote synaptic protein synthesis in rodents, which is crucial for rapid antidepressant responses to ketamine^6^. Therefore, a shorter time may not be sufficient for an antidepressant action of ketamine. The antidepressant effects of ketamine or Yueju appeared to last for 5 or 6 days in depression-like mice, in agreement with sustained antidepressant effects in studies using chronically stressed rats or mice^10^ as well as some clinical findings with ketamine^8^,^36^.

Activation of mTOR signaling in PFC is crucial for rapid antidepressant effects^6^. In the current study, mTOR signaling via 4E-BP1 and p70S6K was suppressed in the PFC of CMS mice, consistent with previous results in CMS rats^17^,^37^,^38^. For the first time, we showed that a single dose of ketamine or Yueju reversed the effects of CMS on the activation of mTOR effectors 4E-BP1 and p70S6K in the PFC of depression-like rodents. Ketamine transiently stimulated mTOR signaling via p-mTOR, 4E-BP1 and p70S6K in PFC of non-stressed rats^6^. Similar effects were also observed after administration of serotonin 2C receptor antagonists that exhibited fast-onset antidepressant effects. Our findings provide new information that in the depression-like mice, ketamine and Yueju induced a sustained increase in phosphorylated 4E-BP1 and p70S6K to the normal level, which may be required for rapid antidepressant responses by promoting synaptic protein synthesis in the PFC of depression-like animals. Particularly, our study suggested that the sustained depression-like behavior was more closely linked with the prolonged deficits in 4E-BP1, whereas p70S6K level recovered at 6 days post termination of stress exposure.

Although chronic stress led to deficits in the activation of mTOR downstream effectors 4E-BP1 and p70S6K, there was no change in the activation of mTOR in the PFC, consistent with previous findings in chronically stressed rats^17^. In contrast, deficits in ERK and Akt activation were found in the chronically stressed mice in the present and previous studies^39^. ERK and Akt both are capable of regulating 4E-BP1 or p70S6K in a manner independent of activation of mTOR^40^. Inhibitors of either ERK or Akt blunted ketamine’s antidepressant effect^6^, plausibly by suppressing 4E-BP1 and p70S6K activation. The deficits in ERK and Akt may suppress 4E-BP1 and p70S6K activation in the chronically stressed animals. Additionally, increased ERK and Akt activity was consistent with increased 4E-BP1 and p70S6K activity resulting from ketamine or Yueju treatment. Interestingly, ERK activation returned to normal level at PAD 6, whereas Akt signaling remained deficient. Normalization of Akt paralleled the antidepressant effect of Yueju, indicating that signaling regulated by Akt pathway may be crucially involved in maintaining depression-like status and antidepressant effects. An increasing number of studies indicate the requirement of Akt signaling in antidepressant effects of ketamine and some conventional antidepressants in non-stressed animals^6^,^41^,^42^. It is of particular interest to further investigate mechanisms by which improvement of Akt signaling mediates antidepressant efficacy in mTOR-dependent and –independent manner.

Some of other NMDA receptor antagonists are also capable of producing rapid antidepressant effects experimentally^9^,^43^. This suggests a role for NMDA systems in the pathology and therapy of depression. We found a significant increase in NR1 subunit of NMDA receptor but not in other two subunits NR2A and NR2B in the PFC of CMS mice. In fact, ketamine, as a noncompetitive NMDA receptor antagonist, acts at the phencyclidine site in NR1 subunit. As NR1 binds both to NR2A and NR2B, our finding suggests a non-selective up-regulation of NMDA activity in the PFC of depression-like mice. Increased expression of NR1 and/or NR2A/NR2B in depressed subjects has been reported previously^44^,^45^. However, other studies indicate down-regulation or no changes^46^,^47^. Differences in experimental results may be due to heterogeneity within MDD patients, different disease stages of MDD, differences in chronic stress procedures, animal genetic backgrounds or regional differences in brain NMDAR subunit expression patterns. It has been shown that blocking NMDA but not AMPA receptors attenuates dendrite atrophy, suggesting that the NMDA receptor is particularly responsible for stress-induced synaptic loss^48^,^49^. A recent study also supports that activation of NMDA (but not AMPA) receptors is required for abolishment of synaptic plasticity and destruction of dendritic spines through the stress-released neuropeptide corticotropin-releasing hormone^50^. Therefore, the enhanced NR1 expression by chronic stress may contribute to sustained NMDAR-mediated dentritic atrophy and spine loss underlying depression-like responses.

Additionally, we found the selective decrease in NR1 by ketamine and Yueju, in contrast to the selectively increased NR1 in the chronically stressed mice. The reduction of NR1 expression, together with the inhibition of NMDA transmission, may result in a strong suppressing effect on NMDA neurotransmission by ketamine and/or Yueju. NR1 expression was also reduced by MK801, an NMDA antagonist similar to ketamine that also shows rapid antidepressant effects^9^. The markedly reduced expression of NR1 has been observed after chronic administration of selective serotonin and norepinephrine uptake inhibitors^51^. The important role of NR1 in the antidepressant effect was especially evident by the finding that inhibition of NR1 was associated with antidepressant effects at 6 days post Yueju administration, whereas ketamine did not reverse NR1 expression and no longer showed antidepressant effects at this time point. It has yet to be elucidated how chronic stress up-regulates NR1 and the molecular mechanisms underlying down-regulation of NR1 by ketamine and Yueju.

Here we found persistent deficits in GluR1 expression. Consistent with our findings, stress enhanced NMDAR and reduced AMPAR activities^52^. A concurrent increase in NR1 and decrease in GluR1 after exposure to chronic stress may indicate an imbalance of AMPAR and NMDAR activities by favoring NMDA throughput. The AMPA receptor subunit GluR1 expression was up-regulated shortly after acute ketamine administration, and lasted for several days in chronically stressed rats, in an mTOR signaling-dependent manner^6^. Here we found a similar up-regulation of GluR1 in the chronically stressed mice post ketamine or Yueju, which can last for an extended time period. Although AMPAR activities apparently are not required for mediating stress induced dendritic atrophy, GluR1 containing synapses are resistant to stress^50^. Increased expression of GluR1 and decreased expression of NR1 at 2 and 6 days post acute administration of Yueju or 2 days post ketamine may increase the ratio of AMPAR/NMDAR activity, in agreement with the findings from chronic ketamine administration^25^. These results support the idea of the association between increased AMPAR/NMDAR ratios and antidepressant responses^23^. Increasing the ratio of AMPAR-to-NMDAR activities by favoring AMPAR throughput may represent an enhanced capability of glutamate machinery to counteract adverse consequences of stress.

**Figure 6:**
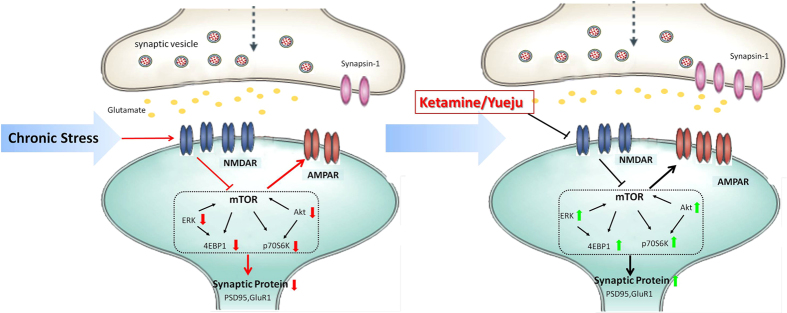
A working model of the effects of fast-acting antidepressants on mTOR related activity and AMPA/NMDA receptor expressions in the PFC of a rodent model of depression. Chronic stress promotes NMDAR activities that mediate synaptic loss. Up-regulated NMDAR activities decrease Akt and ERK signaling, which in turn suppresses activities of mTOR downstream effectors p70S6K and 4E-BP1, leading to decreased synaptogenesis and dendrite atrophy, including decreased AMPAR activities. Consequently, the ratio of AMPA/NMDA receptor function decreases, contributing to depression-like behaviors. Yueju and ketamine attenuate the up-regulated NMDAR expressions and/or inhibit NMDA neurotransmission in chronically stressed subjects. The substantially reduced NMDA activities improve the Akt and ERK signaling and downstream signaling of p70S6K and 4E-BP1, leading to increased synaptogenesis and AMPAR activities. Therefore, the ratio of AMPA/NMDA receptor function increases, contributing to rapid antidepressant effects in depression-like subjects. The diagram was drawn by Juanjuan Tang according to the present experiment.

The current study is the first to demonstrate that changes in mTOR-related activity and glutamate receptors caused by chronic stress can be reversed by two distinct rapid antidepressants, ketamine and Yueju. We confirmed that ketamine and Yueju acted as quickly as 2 hours post-drug administration and result in a long-lasting reduction in depression-like symptoms by using a comprehensive behavioral test battery in a chronic model of depression. Based on our findings, a framework for the pathological changes in the PFC after exposure of chronic stress and molecular therapeutic mechanisms is proposed in Fig. 6. We show that a single dose of ketamine or Yueju reversed deficits in mTOR-related signaling, synaptic protein synthesis as well as the imbalance of AMPA/NMDA receptor ratio in the PFC of CMS mice, contributing to the fast-acting and lasting antidepressant effects. This study sheds the new light on targeting pathological changes in glutamate neurotransmission and mTOR-related signaling in depressed subjects using fast-acting antidepressants.

## Methods

### Animals

Male Kunming mice^53^ were purchased from the China Academy of Military Medical Sciences (Beijing). All animal procedures were carried out in accordance with the Guide for the Care and Use of Laboratory Animals approved by the Institutional Animal Care and Use Committee at Nanjing University of Chinese medicine. Mice aged 6–8 weeks old were group housed in standard shoebox cages for 1 week before experimental procedures. The experimenters were blind to the assignments of the mice to the experimental groups.

### Drugs

Ketamine HCl (30 mg/kg, Gutian Pharmaceuticals, China) and fluoxetine (15 mg/kg, Sigma) was administered intraperitoneally (i.p.) as a 1 mg/ml in saline concentration. The preparation of Yueju ethanol extract and the quality control using HPLC followed the protocol of Xue *et al.*^14^. The medicinal plants used to prepare Yueju include: Cyperus rotundus (Xiang Fu), Ligusticum Chuanxiong (Chuan Xiong), Gardeian jasminoides (Zhi Zi), Atractylodes lancea (Chang Zu) and Massa Fermentata (Shen Qu), in which oil fraction of Gardeian jasminoides showed potent antidepressant-like effects, which contained 16 compounds including myristic acid and 2,4-Decadienal^28^. Moreover, only the ethanol extract of Gardeian jasminoides conferred rapid antidepressant effects, whereas other medicinal plants may play a supporting role^54^. The medicinal plants were purchased from Nanjing Guoyi Clinical, Medicinal Material Department (Nanjing, China). Briefly, the herb mixtures were powdered, immersed in 95% of ethanol with constant shaking, and filtered. This procedure was repeated three times, and the collected solvent was evaporated at low pressure and medium temperature (<55 °C) until ethanol was completely eliminated. The extract of Yueju was dispersed in Tween 80 solution (0.5%, w/v in saline) and administrated intragastrically (270 mg/ml, i.g.). The doses of ketamine and Yueju were optimized based on a pilot assessment of a dose-response relationship in the strain of mice (Supplement 1). 0.5%, w/v Tween 80 in saline solution via i.g. and saline via i.p served as the vehicle controls, and their behavioral data or Western blot were collapsed as there were no statistical differences between them.

### Chronic Mild Stress (CMS) Paradigm

The CMS procedure was done as described previously^55^ with minor modifications. Mice were singly housed and received 3 weeks of mild stress, with the stressors as follows: restraint in a 50 ml tube for 6 hours, cage tilted for 45° for 24 hours, cage shaking at high speed (200 rpm) for 40 minutes, water deprivation for 24 hours, food deprivation for 24 hours, soiled bedding (200 ml) for 20 hours, and overnight illumination (12 hours). Mice received a stress daily with the order of the stressors changing weekly, except that overnight illumination was administrated twice a week, 3–4 days apart. Naïve mice were group-housed under normal conditions.

The day after the last stressor was given, a single dose of individual drugs was administrated. Three independent cohorts were tested at different times post drug administration (Table 1). In all three cohorts, CMS mice received vehicle, Yueju, and ketamine, whereas naïve mice received vehicle. In cohort #1, an additional CMS group received acute fluoxetine, and all animals were only tested for sucrose preference at 2 hours post drug administration. In cohort #2, at 1 day post drug administration, a battery of behaviors were assessed in the same animals in the following order, using the procedure of Zhang *et al.*^55^: open field test, tail suspension test, and forced swim test, with at least 2 hours between test sessions. Novelty-suppressed feeding test and sucrose preference test were carried out on the following morning. In this battery testing procedure, the prior test or tests in the prior day did not induce noticeable impact on the subsequent tests based on a pilot observation. Same procedure was repeated in cohort #3, except that the testing started at 5 days post drug administration. Group of fluoxetine was not included in the Cohorts #2 and #3 as the results of cohort #1 indicated no effect of a single dose of fluoxetine on the chronically-stressed animals. Animals were weighted before treatment and on the last day of test.

## Behavioral Testing

### Open Field Test (OFT)

The OFT assesses locomotor activity and anxiety-like behavior in a bright-lit open area. Testing was performed in a well-illuminated (~300 lux) transparent acrylic cage (40 × 40 × 15 cm). The mice were gently placed in the center and left to explore the area for 5 minutes. The digitized image of the path taken by each mouse was tracked by camera, and the total running distance (locomotor activity) and time spent in the center were analyzed using ANY-maze software. Each mouse was placed in the middle of a peripheral zone of the arena facing the wall and allowed to freely explore the apparatus, with the experimenter out of the animal’s sight.

### Tail Suspension Test (TST)

TST is one of the most widely used paradigm for assessing factors that influence depression-like behavior (behavioral despair) and antidepressant response in mice (Steru et al., 1987)56. TST was performed using a computerized system which allowed four animals to be videotaped and tested at one time. In a chamber both acoustically and visually isolated, an individual mouse was suspended by the tail 50 cm above the floor using adhesive tape placed approximately 1 cm from the tip of the tail. The total duration of the test (6 minutes) can be divided into periods of agitation and immobility. Total immobility times during the last 4 minutes of a 6-minute-test were calculated by ANY-maze software. No animal climbed its tail during the test.

### Forced Swim Test (FST)

FST is also a widely used paradigm for behavioral despair and antidepressant response in rodents. FST was carried out as reported^57^. Mice were removed from their home cages, placed individually into a clear glass tank (40 cm high and 20 cm in diameter) filled with 30 cm of water (22–23 °C) and allowed to swim for 6 minutes^58^. At the end of the test, the animals were removed from the water, dried with a paper towel and returned to their home cages. The mice were considered immobile when floating in the water without struggling and making only those movements necessary to keep their heads above the water. Total immobility times during the last 4 minutes of the 6 minute testing period were recorded by ANY-maze software.

### Novelty-suppressed feeding (NSF) test

The NSF was designed to assess the anxiolytic and/or antidepressant effects of chronic antidepressant treatment in rodents^59^. The testing apparatus consisted of a plastic box that measured 50 × 50 × 20 cm. At 18 hours prior to behavioral testing, all food was removed from the rodent’s home cage. At the time of testing, a weighed single pellet of normal food chow for mice was placed on a white paper platform positioned in the center of the plastic box. Each mouse was placed in a corner of the box and allowed to explore for up to 10 minutes. The trial ended when the mouse chewed a part of the chow. The amount of food consumed in the home cage was taken as the weight of chow consumed in 10 minutes. The time first mouse started to consume the food pellet was recorded as the latency.

### Sucrose Preference Test (SPT)

The SPT followed a published procedure with minor modifications^60^. Briefly, mice were individually housed and exposed to a sucrose solution (1% in tap water, Sigma, St Louis, MO, USA) for 72 hours, followed by 18 hours of water deprivation and a 2 hour exposure to two identical bottles, one filled with 1% sucrose solution and the other with water. The volumes of sucrose solution and water were measured for the 2 hour exposure. Sucrose preference was defined as the ratio of the volume of sucrose versus total volume (sucrose + water) consumed during the 2 hour test, normalized to each individual animals’ body weight.

### Western Blot

The whole prefrontal cortex was lysed in RIPA buffer containing protease and/or phosphatase inhibitors. Protein concentration was determined colorimetrically by BCA assay (Pierce, Rockford, IL, USA). Protein lysates were separated by SDS-PAGE electrophoresis and were transferred onto polyvinylidene difluoride (PVDF) membranes. Antibodies for Western blots were from Cell Signaling Technologyunless indicated. Product numbers and antibody dilutions are indicated within parentheses. After blocking with 5% BSA for 1 hour, the membranes were incubated with NR1 (#5704, 1:1000), NR2A (#4205, 1:1000), NR2B (#4212, 1:1000), PSD-95 (Millipore, #04-1066, 1:1000), GluR1 (#13185, 1:1000), synapsin-1 (Abcam, ab18814, 1:1000), p-mTOR (#2971, 1:1000), mTOR (#2972, 1:1000), phosphor-4EBP-1 (#2855, 1:1000), phosphor-p70S6K(#9324, 1:1000), p70S6K(#2708, 1:1000), phosphor-Akt (#4060, 1:1000), Akt (#9272, 1: 1000), phosphor—ERK(#4370, 1:1000), ERK (#9120, 1:1000)and β-Tubulin (Abcam, ab151318, 1:2000) antibodies at 4 °C overnight, followed by incubation with the appropriate horseradish peroxidase-conjugated secondary antibody for 1 hour. Then, the blots were visualized using the SuperSignal West Pico Chemiluminescent Substrate (Thermo Fisher Scientific Inc.). For analysis, NR1, NR2A, NR2B, p-4EBP-1, PSD-95, synapsin-1 and GluR1 were normalized to β-Tubulin bands, and p-mTOR, p-Akt, p-ERK and p-p70S6K bands were normalized to total protein levels then expressed as a percentage of that in control animals. For figure panels, contrasts have been adjusted linearly for easier viewing of bands. All experiments were performed in quadruplicate.

### Statistical Analyses

Comparisons were made using a one-way analysis of variance (ANOVA), followed by the *post hoc* comparisons with Bonferroni method. Analyses of variance with repeated measures were used for body weight comparison, and Bonferroni *post hoc* tests examined the changes from pre-drug treatment at each time point as well as group differences. All data are presented as mean ± SEM, and statistical significance was accepted at the 5% level unless otherwise indicated.

## Additional Information

**How to cite this article**: Tang, J. *et al.* Involvement of normalized NMDA receptor and mTOR-related signaling in rapid antidepressant effects of Yueju and ketamine on chronically stressed mice. *Sci. Rep.* **5**, 13573; doi: 10.1038/srep13573 (2015).

## Supplementary Material

Supplementary Information

## Figures and Tables

**Table 1:** Three independent cohorts of animals were used to test the rapid and long-lasting antidepressant effects of ketamine and Yueju on CMS mice.

	Vehicle	CMS + Vehicle	CMS + Yueju	CMS + ketamine	CMS + fluoxetine
cohort#1	SPT at 2 hours post drug administration				
cohort#2	OPT,TST,FST at 1 day, NSF,SPT at 2 day post drug administration				
cohort#3	OPT,TST,FST at 5 day, NSF,SPT at 6 day post drug administration				
