# Supplementary material for: Involvement of normalized NMDA receptor and mTOR-related signaling in rapid antidepressant effects of Yueju and ketamine on chronically stressed mice
Source: Sci Rep. 2015 Aug 28;5:13573. doi: 10.1038/srep13573 (PMC4551989; doi:10.1038/srep13573)
Supplement: Supplementary Information [file srep13573-s1.docx]

**Involvement of normalized NMDA receptor and mTOR-related signaling in rapid antidepressant effects of Yueju and ketamine on chronically stressed mice**

**Juanjuan Tang^123^****^#^,Wenda Xue^12^****^#^, Baomei Xia^12^, Li Ren^12^, Weiwei Tao^12^, Chang Chen^4^, Hailou Zhang****^12^, Ruyan Wu^12^, Qisheng Wang^12^, Haoxin Wu^2^, Jinao Duan^12^, Gang Chen^12*^**

^1^ Center for Translational Systems Biology and Neuroscience, School of Basic Biomedical Science, Nanjing University of Chinese Medicine, Nanjing 210023, China

^2^Key Laboratory of Integrative Biomedicine of Brain Diseases, School of Basic Biomedical Science, Nanjing University of Chinese Medicine, Nanjing 210023, China

^3^Physiology Research Section, School of Basic Biomedical Science, Nanjing University of Chinese Medicine, Nanjing 210023, China

^4^ First Clinical Medical College, Nanjing University of Chinese Medicine, Nanjing 210023, China

^#^These authors contributed equally to this work.

E-mail: chengang@njucm.edu.cn

**Inventory of Supplementary Information**

**Supplementary Figure**

Supplementary Figure S1

Supplementary Figure S2


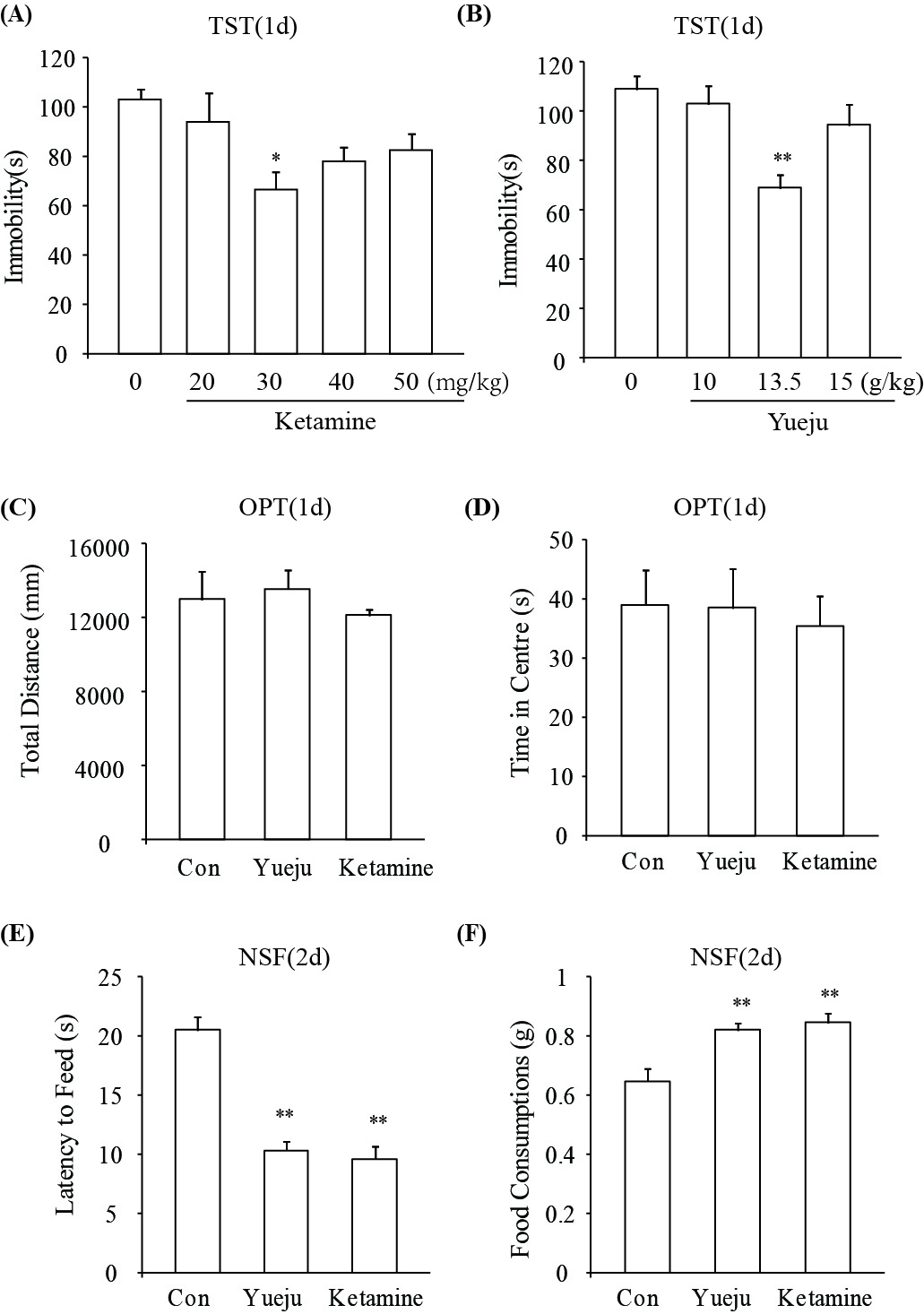


**Supplementary Figure S1** The doses of ketamine and Yueju were optimized based on a pilot assessment of a dose-response relationship in the strain of mice. (A) The immobility time in the TST 1 day after different concentration of ketamine administration. ANOVA, F(5,59)=3.584, p<0.05. (B) The immobility time in the TST 1 day after different concentration of Yueju administration. ANOVA, F(3,47)=6.939, p<0.05. (C) In the open field test, there was no significant difference on either total distance traveled (ANOVA, F(2,17)=0.449, p=0.647) or (D) the time spent on the central zone (ANOVA, F(2,17)=0.104, p=0.902) 1d after ketamine and Yueju administration. (E) Latency to feed in the NSF test at 2 days after ketamine and Yueju administration, ANOVA, F(2,23)=35.264, p<0.05. (F) Total amount of food consumed in the NSF test at 2 days after ketamine and Yueju administration, ANOVA, F(2,23)=9.867, p<0.05 in the novelty-suppressed feeding test.* p<0.05, ** p<0.01,compared with control group, and n=6-12.


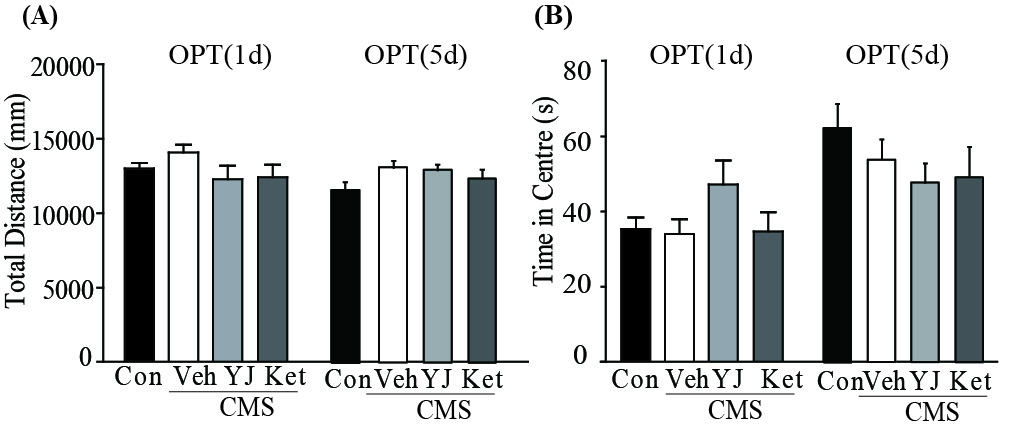


**Supplementary Figure S2** In the open field test, there was no significant difference on either total distance traveled, a measurement of locomotor, 1d, ANOVA, F(3,31)=1.472, p=0.244, 5d, ANOVA, F(3,37)=1.607, p=0.206 or the time spent on the central zone, a measurement of anxiety, 1d, ANOVA, F(3,31)=1.194, p=0.33, 5d, ANOVA, F(3,37)=1.032, p=0.391, n=7-10.
